# The development of a hairless phenotype in barley roots treated with gold nanoparticles is accompanied by changes in the symplasmic communication

**DOI:** 10.1038/s41598-019-41164-7

**Published:** 2019-03-18

**Authors:** Anna Milewska-Hendel, Weronika Witek, Aleksandra Rypień, Maciej Zubko, Rafal Baranski, Danuta Stróż, Ewa U. Kurczyńska

**Affiliations:** 1 Department of Cell Biology, Faculty of Biology and Environmental Protection, University of Silesia in Katowice, 28 Jagiellońska Street, 40-032 Katowice, Poland; 2 Laboratory of Microscopy Techniques, Faculty of Biology and Environmental Protection, University of Silesia in Katowice, 28 Jagiellońska Street, 40-032 Katowice, Poland; 3 Institute of Materials Science, Faculty of Computer Science and Materials Science, University of Silesia in Katowice, 75 Pułku Piechoty Street 1a, Chorzów, 41-500 Poland; 4 Department of Physics, University of Hradec Králové, Hradec Králové, Czech Republic; 5 Institute of Plant Biology and Biotechnology, Faculty of Biotechnology and Horticulture, University of Agriculture in Krakow, Al. 29 Listopada 54, 31-425 Krakow, Poland

## Abstract

Uptake of water and nutrients by roots affects the ontogenesis of the whole plant. Nanoparticles, e.g. gold nanoparticles, have a broad range of applications in many fields which leads to the transfer of these materials into the environment. Thus, the understanding of their impact on the growth and development of the root system is an emerging issue. During our studies on the effect of positively charged gold nanoparticles on the barley roots, a hairless phenotype was found. We investigated whether this phenotype correlates with changes in symplasmic communication, which is an important factor that regulates, among others, differentiation of the rhizodermis into hair and non-hair cells. The results showed no restriction in symplasmic communication in the treated roots, in contrast to the control roots, in which the trichoblasts and atrichoblasts were symplasmically isolated during their differentiation. Moreover, differences concerning the root morphology, histology, ultrastructure and the cell wall composition were detected between the control and the treated roots. These findings suggest that the harmful effect of nanoparticles on plant growth may, among others, consist in disrupting the symplasmic communication/isolation, which leads to the development of a hairless root phenotype, thus limiting the functioning of the roots.

## Introduction

Although the effect of nanoparticles (NPs) on plant growth is well documented^1–3^, we are still far from full understanding of mutual interactions between NPs and the developmental processes, in particular, the mechanisms that lead to reduction in plant growth under their influence.

The root system is the organ that is responsible for water and nutrient uptake from the soil^4–7^. One developmental strategy that is favoured by plants is to increase the root-soil contact *via* the development of root hairs that enhance water and nutrient uptake^8^. The root epidermis is composed of cells that produce root hairs (which are derived from trichoblasts) and non-hair cells (which are derived from atrichoblasts)^9,10^. These cells begin to differentiate from tricho- and atrichoblasts in the differentiation zone of a root^11^. Thus, the development of trichoblasts increases the surface that is involved in the uptake of water and nutrients. Any disorder in the development of trichoblasts leads to limitation of the nutrient uptake, which is necessary for normal plant development.

The evaluation of the effect of NPs on plants is extremely important because some metal nanomaterials are considered to be nano-fertilizers (NFs). NF balances the release of mineral nutrients with the absorption by the plant, thus they can increase the efficiency of nutrient use^12,13^. Therefore, NFs may improve crop productivity by enhancing the rate of seed germination, plant growth, photosynthetic activity or nitrogen metabolism^14^. Although nanotechnology has incredible potential in the agricultural sector, it may have unknown risk due to their environmental and health impact that can prevail over their potential benefits^15^. The evaluation of these risks is associated with the new field of knowledge, “nanotoxicology,” which confirms the need to analyse the influence of nanomaterials on living organisms^16^.

Hitherto, numerous literature results have indicated that direct exposure to different types of NPs may have a phytotoxic effect on root growth and development. Morphological, cellular and molecular alterations have been observed in wheat roots under the influence of aluminum oxide nanoparticles^17^. An inhibition of root hair development was also observed in Arabidopsis seedlings that were grown in the presence of different NPs^18^ as well as in the roots of rice that had been treated with the AgNPs^19^. Despite the increasing number of reports about the NPs-plant interactions, our knowledge about the influence of NPs on the development and growth of root hairs is still insufficient and no general conclusion can be drawn.

One aspect has not previously been studied in relation to the effect of NPs on root hairs, namely the involvement of symplasmic communication in the differentiation of trichoblasts which might be changed by the influence of NPs. It is well documented that the isolation of symplasmic communication is one of the mechanisms that is involved in cell differentiation during both the embryogenic and postembryogenic stages of development^20–29^. At present, our knowledge about the correlation between symplasmic communication and root development, indicates that the specification of cell fate and organ root formation depends on the movement of molecules through the plasmodesmata (PDs)^20,30^.

It is known that the differentiation of trichoblasts and atrichoblasts is accompanied by a restriction of symplasmic communication between cells that are undergoing two different developmental programmes^10^. It was already showed for barley that symplasmic communication was diversed in hairless mutants and a parental variety^31^. Namely, in the root hairless mutants the epidermal cells were symplasmically connected in a differentiation root zone, thus leading to a lack of differentiation of the cells into hair and non-hair cells^26,31^.

The symplasmic movement of molecules occurs through the PDs^26,32,33^. While PDs are the intracellular channels for the diffusion of small molecules such as ions and/or sugars, they are also dynamic gateways that actively transport or block the cell-to-cell transport of macromolecules such as proteins, including the transcriptional factors and RNAs including miRNA or siRNA^26,34–36^. Among the transcriptional factors that are involved in regulating cell differentiation, some of them also participate in root hair development, e.g. TRANSPARENT TESTA GLABRA1^37^, CAPRICE^38^ or CAPRICE-LIKE MYB3^39^.

During our studies on the effect of positively charged gold nanoparticles (AuNPs) on the growth of barley seedlings, we found out that the roots of plants that were growing in a medium enriched with NPs were hairless. Moreover, we observed alterations on the morphological, histological and ultrastructural level as well as the changes in cell wall composition in the treated roots. Thus, the aim of this study was to determine whether the appearance of various stress responses in the barley roots treated with AuNPs are accompanied by changes in the distribution of the symplasmic tracers. This may suggest that the described negative effect of NPs on plant growth may also be the result of the remodeling of the symplasmic connectivity of the rhizodermal cells which block the differentiation of the root hair cells, thereby leading to a limitation in the water and nutrient absorption by the root system.

## Results

### Morphological and histological changes in the roots treated with positively charged AuNPs

**Figure 1:**
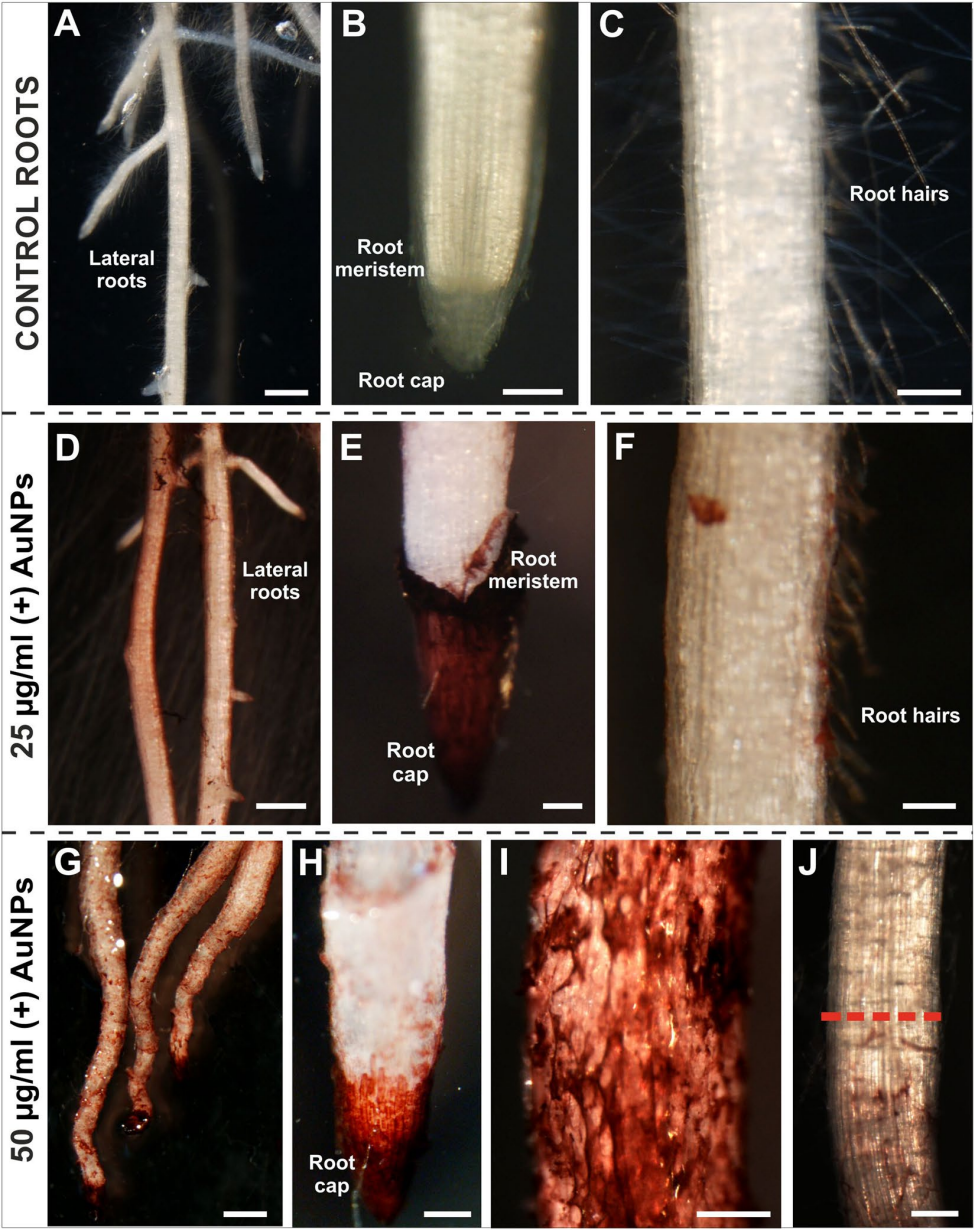
Morphology of the barley roots: control (**A**–**C**), treated with AuNPs at a lower concentration (**D–F**) and at a higher concentration (**G**–**J**). The roots from the control plants developed lateral roots (**A**) and numerous root hairs (**C**). The meristematic part of the root had no symptoms of decay (**B**). The root apical meristem was covered with the root cap. The treated roots were reddish in colour, regardless of the concentration (**D–J**). The most intense red colour characterised the root cap (**E**,**H**), but the colouration was also visible along the entire length of the root surface that remained in contact with the solution (**D**,**G**). After treatment with the high concentration of AuNPs, the roots were curved (**G**). The part of the root above the AuNPs solution was not coloured (**J**, above the red dotted line; control = 7 biological replicas; 90 roots in total; 25 µg/ml AuNPs = 5 biological replicas, 60 roots in total; 50 µg/ml AuNPs = 6 biological replicas, 87 roots in total). Scale bars: A, D, G = 1 mm; B,C,E,F,H,I,J = 200 µm.

The morphology of the roots from the seven-day-old control seedlings was significantly different from that of the plants that have been treated with nanogold (Fig. 1A–J) especially compared to those that were growing in the medium with a high concentration of AuNPs (50 μg/ml). The first developmental sign was the emergence of the radicle, which in the case of the control plants, developed lateral roots (Fig. 1A). The morphology of the control roots revealed that the phenotype was similar to one that had been described earlier^31^. Four zones were detected along the root longitudinal axis: meristematic (Fig. 1B), elongation, differentiation (Fig. 1C) and the lateral root zone (Fig. 1A). The last of the above-mentioned zones could not be identified in the roots that had been treated with AuNPs at a high concentration (Fig. 1G). However, the roots that had been treated with AuNPs at a lower concentration (25 μg/ml) developed lateral roots (Fig. 1D) and most of them developed root hairs (Fig. 1F). The surface of the treated roots was reddish regardless of the concentration (Fig. 1D–J) and the most intensive colouration was visible on the root tip, especially in the area of the root cap (Fig. 1E,H). The morphology of the roots from the seedlings that were growing in a high concentration was completely changed; the roots were bent (Fig. 1G) and they did not develop root hairs or lateral roots (Fig. 1I,J), even when their surface was above the solution level (Fig. 1J). In the control goup, all roots had root hairs, however at the 25 μg/ml AuNPs concentration the frequency of roots with hairs was lower (75.7%; *P* = 0.002) and at 50 μg/ml AuNPs roots with hairs were very rare (11.1%; *P* = 0.001) (Table S1).

**Figure 2:**
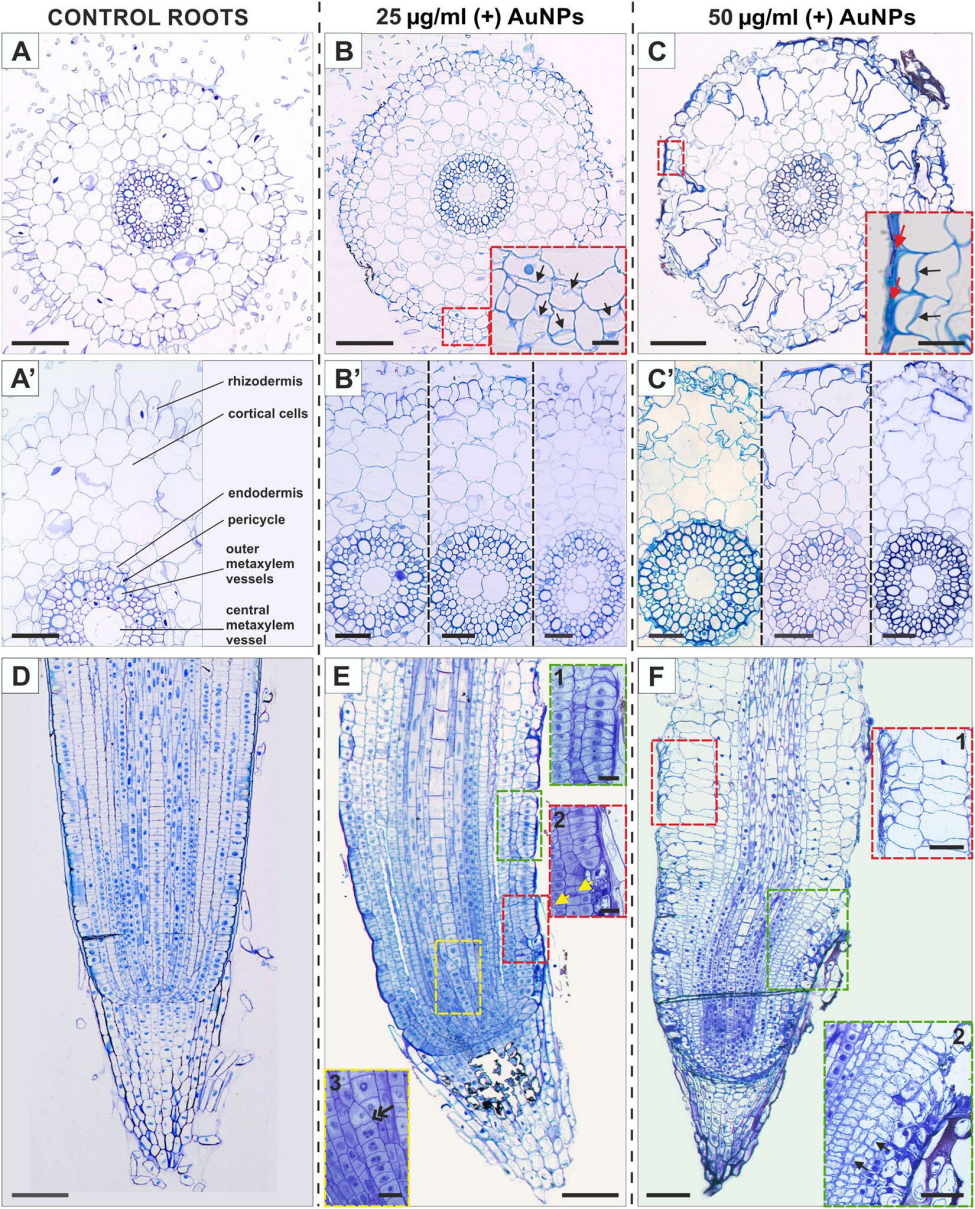
Cross sections through the differentiation zone (**A–C**′) and longitudinal sections through the root tip (**D–F**) of the control (**A,A′,D**) and AuNPs-treated at a lower concentration (**B**,**B′,E**) and a higher concentration (**C,C′,F**) roots of the barley seedlings. The histology of the roots from the control plants was typical for barley and consisted of hair and non-hair cells; the cortical cells were formed by four layers; the endodermis and pericycle were well visible; the central vascular cylinder was composed of meta- and protoxylem vessels and phloem poles (**A,A′**). The same tissues were present in the treated roots (**B,B′,C,C′**). In the roots that had been treated with a lower concentration of AuNPs, atypical periclinal divisions were observed (**B** inset, black arrows; **E** inset 2, yellow arrows). A multiplication of the central metaxylem vessel (**B,B′**) and its division probably occurred in close proximity to the root tip (**E** inset 3, double arrow). Another alteration was the formation of multicellular packets of cortical and rhizodermal cells (**E** inset 1). In the roots that had been treated with a high concentration of AuNPs, all of the rhizodermal cells displayed the same phenotype (**C,C′**). There was a visible thickness of the outer periclinal cell wall of rhizodermis (**C** inset, red arrows). The longitudinal section exhibited prominent alterations in the cell arrangement especially in the layers of the cortical cells (**F**) whose dimensions increased (**F** inset 1). Atypical divisions of the rhizodermal and cortical cells were also observed (**F** inset 2, black arrows). The sections were stained with TBO. Each section is representative of five biological replicas; in total 45 roots were analysed histologically. Scale bars: A,B,C,D,E,F = 100 µm; A′,B′,C′,C inset, F insets = 50 µm; A,C,E insets = 20 µm.

The same types of tissues were observed on the cross sections through the differentiation zone (Fig. 2A–C′) and on the longitudinal sections through the root tip of the treated and control roots (Fig. 2D–F). The rhizodermis of the control roots consisted of root hair and non-hair cells, four layers of cortical cells, the endodermis, pericycle and a vascular bundle were also present (Fig. 2A,A′). The changes in the meristematic zone size and pattern between the control and treated roots are presented on longitudinal sections of root tips (Fig. 2D–F). The histological alterations in the roots that had been treated with a lower concentration of AuNPs revealed atypical periclinal divisions of the rhizodermal and cortical cells (Fig. 2B inset, black arrows; 2E inset 2, yellow arrows) and a multiplication of the central metaxylem vessel (Fig. 2B,B′). This multiplication probably began in close proximity to the root tip which is visible as an oblique cell division on the longitudinal section (Fig. 2E inset 3, double arrow). Moreover, disruption of regular cell files (Fig. 2E) and the formation of multicellular packets of rhizodermis (Fig. 2E inset 1) and cortical cells were also observed. A higher concentration of positively charged AuNPs caused a disrupted pattern of the rhizodermis. All of the rhizodermal cells presented the same phenotype, and therefore distinguishing between the root hair and non-hair cells was not possible (Fig. 2C,C′). Periclinal divisions were frequently observed in the rhizodermal (Fig. 2C inset, black arrows) and cortical cells (Fig. 2F inset 2, black arrows) and thickness of the outer periclinal cell wall was also observed (Fig. 2C inset, red arrows). A multiplication of the central metaxylem vessel was also observed (Fig. 2C′). Moreover, visible prominent alterations of the cell arrangement was observed on the longitudinal section through the root tip, especially in the layers of the cortex (Fig. 2F) where there was also a significant increase in the radial cell dimensions of the first two layers of the cortical cells (Fig. 2C,F inset 1).

Analysis of root number for the control group and the plants that were growing under the influence of AuNPs did not show statistically significant differences and all the plants developed six roots on average (Table 1). The average root length for the control plants was 3.9 mm, while for the plants that had been treated with lower and higher concentrations of AuNPs were 3.6 mm and 2.9 mm, respectively. The differences between the control plants and those that had been treated with 50 µg/ml AuNPs were statistically significant (Table 1, Fig. S1). The most pronounced differences were related to the cell diameter of different root tissues apart from the non-hair cells (Table 1, Fig. S2; hair cells were not measured because they were not present in the roots that had been treated with the higher concentration of AuNPs; strict criteria for measuring the diameter of root hair cells is impossible due to their high degree of variability, even at the same distance from the top of the root. Therefore, it was deemed that such a result would not provide scientifically significant information). In general, the cell diameter in the roots that had been treated with the higher concentration of AuNPs was significantly larger compared to the control roots and it was the most conspicuous for the two outer layers of the cortex (Table 1, Fig. S2), the cortex with endodermis (Table 1, Fig. S6) and the central cylinder with the pericycle (Table 1, Fig. S7). In the control roots the average length of the hair cells equal to 141.7 µm was smaller than for the non-hair cells (238.5 µm) (Table 1). The length of rhizodermal cells that had been treated with AuNP was smaller (99.5 µm) than that in the control group (Table 1).

**Table 1:** Selected characteristics of the control and AuNPs-treated roots.

Characteristics	ANOVA		Mean values		
	F^a^	*P* ^b^	control	25 µg/ml AuNPs	50 µg/ml AuNPs
Mean root number [n = 15]	1.048	0.417	6.5	5.3	7.1
Mean root length [mm; n = 60]	3.492	0.041	3.91a^c^	3.58ab	2.87b
Mean length of root meristematic zone [µm; n = 25]	2.344	0.123	365.00	393.25	297.00
Mean length of rhizodermal cells [µm; n = 74]^d^	86.213	<0.001	238.53a (non-hair cells) 141.72b (hair cells)		99.53c
Mean cell diameter [µm; n = 47]					
*- non-hair cells*	1.663	0.201	20.09	25.41	21.53
*- 1* ^*st*^ *cortex layer*	9.135	<0.001	27.37b	35.81ab	59.11a
*- 2* ^*nd*^ *cortex layer*	12.639	<0.001	42.79b	59.44ab	72.81a
*- 3* ^*rd*^ *cortex layer*	15.794	<0.001	34.50b	52.33a	55.17a
*- 4* ^*th*^ *cortex layer*	7.902	0.001	25.67b	42.13a	24.30b
*- cortex & endodermis*	24.698	<0.001	144.97b	202.35a	226.45a
*- central cylinder & pericycle*	21.712	<0.001	136.40b	199.30a	183.55 9

^a^F – Fisher test value; ^b^P – significance level; ^c^For characteristics with ANOVA P < 0.05 the differences between means were compared using the Tukey’s honest significant difference (HSD) test; means followed by the same letter do not differ significantly according to the Tukey’s HSD test at P = 0.05; ^d^data for the control and 50 µg/ml AuNPs-treated roots only; n – number of evaluated samples.

### AuNPs treatment affect symplasmic communication in the barley roots

The morphological and histological observations, especially of the hairless phenotype of the roots that had been treated with a higher concentration of AuNP, convinced us to examine the distribution of the symplasmic tracers among the cells of rhizodermis.

Two different symplasmic tracer fluorochromes were used in the analysis. Because HPTS is not a membrane-permeant probe, after its application (which requires membrane damage), its movement between the cells occurs only through the PDs, thereby indicating the presence of symplasmic communication between cells. FDA is a membrane-permeant probe of fluorescein diacetate. FDA is a non-fluorescent probe that is changed into the membrane-impermeant fluorescent fluorescein by the esterases in the cytoplasm, and as in the HPTS probe, its distribution indicates the presence or absence of symplasmic transport^29,31^.

**Figure 3:**
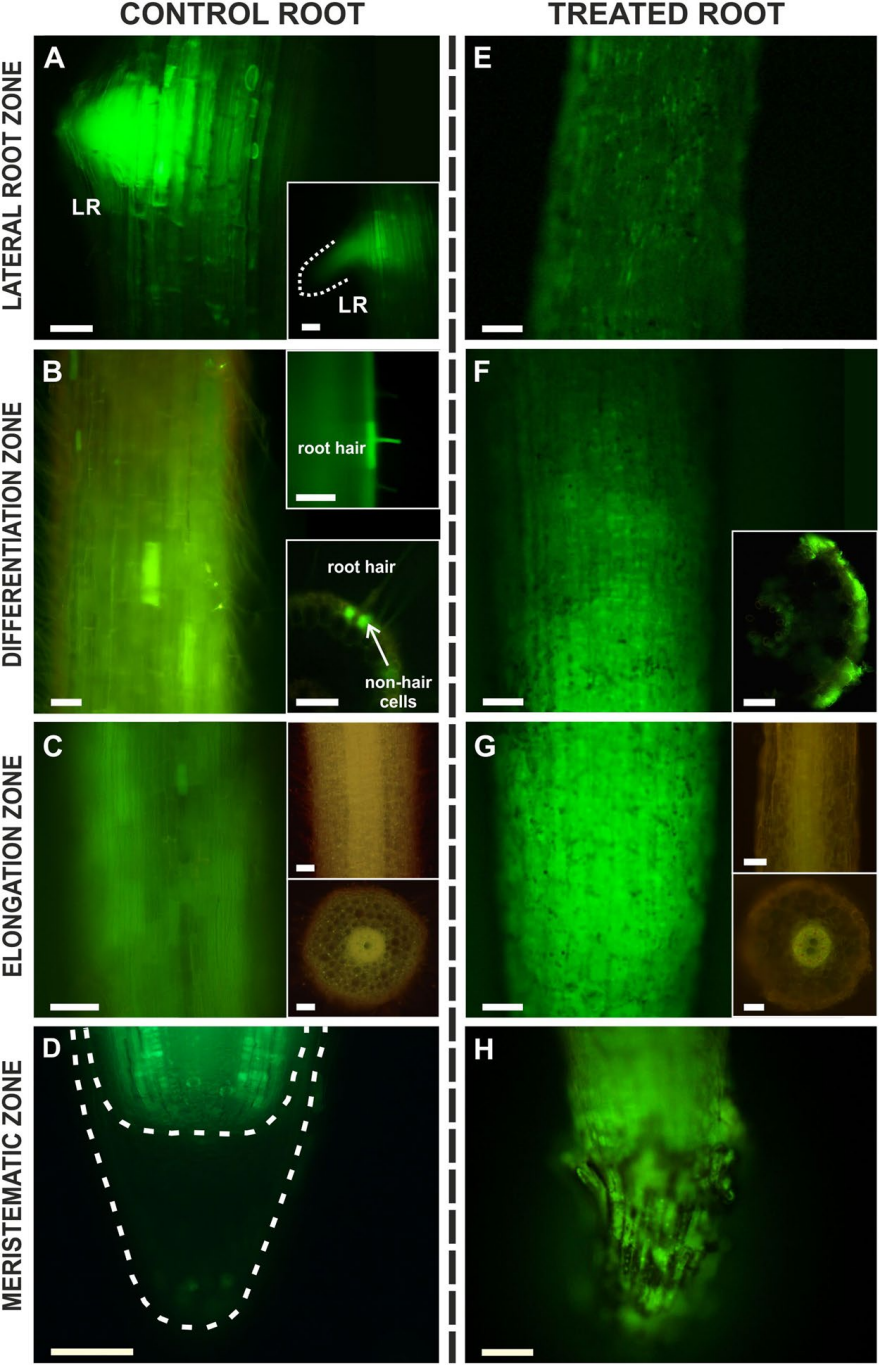
Distribution of HPTS in the control (**A**–**D**) and AuNPs-treated roots (**E**–**H**) along the longitudinal axis and cross hand-cut sections. A higher intensity of fluorochrome was observed in the lateral root (**A**; lack of signal in the root cap in the lateral root – inset; a dotted white line marks the surface of the meristem of the lateral root). In the differentiation zone of the control roots, the presence of fluorochrome was only detected in some cells (**B**; upper inset – fluorochrome was present only in the root hair cells; lower inset – on the cross section, fluorochrome was present in the non-hair cells). In the elongation zone, fluorochrome was only present in some of the rhizodermis cells but in more cells compared to the differentiation zone (**C**; insets show the autofluorescence of the roots surface – upper inset, and on cross section – lower inset). In the meristematic zone, fluorochrome was present in some of the cells and no signal was detected in the root cap (**D**; dashed white lines indicate the surface of the root cap and the border between the root and root cap). In the AuNPs-treated roots (**E**–**H**), an intensive signal was detected in all of the root zones (**F**–**H**) except for the zone (**E**) corresponding to the lateral root zone in the control roots (determined on the basis of the distance from the apex although due to the differences in the length of the cells, it is quite relative) where the signal was less intensive (**E**). The most intensive signal was observed in the cells in the elongation zone (**G**; insets show the autofluorescence of the roots surface – upper inset, and on cross section – lower inset for treated roots). Each section is representative of five biological replicas; in total 45 roots were analysed. Scale bars = 100 µm.

The symplasmic communication along the longitudinal axis of roots is presented in representative figures that are based on the movement of HPTS (Fig. 3A–H). The application of fluorochrome to the basal part of the root was performed in order to determine which tissues are involved in fluorochrome distribution, thereby indicating between which tissues symplasmic communication occurred. In the roots from the control seedlings, the fluorescence signal was present along the entire length of a root (Fig. 3A–D). No connection between the root apical part and the root cap was detected with this fluorochrome (Fig. 3D). The distribution of a fluorochrome within the rhizodermal cells had a patchy character especially in the elongation and differentiation zones (Fig. 3B,B insets; C). The observations revealed that the root hair and non-hair cells are symplasmically isolated as they did not exhibit any distribution of HPTS to the neighbouring cells (Fig. 3B insets). Analysis of the fluorochrome distribution in the lateral roots indicated that a symplasmic connection exists between the vascular bundle of the main roots and the lateral ones (Fig. 3A).

An intensive signal in the rhizodermal cells was detected in the meristematic and elongation zones in the AuNPs-treated roots, and a less intensive signal was observed in the differentiation zone and “lateral root zone” (Fig. 3E–H, F inset; as was mentioned above, different root zones could not be identified in the treated roots using morphological features such as the presence of root hairs or lateral roots. However, these regions were arbitrarily determined taking into account the distance from the apex and the length of the rhizodermal cells). In the root cap cells from the treated roots, an intensive fluorescence signal was detected comparing to the control roots (Fig. 3D,H). In the corresponding differentiation zone, the fluorochrome in the control roots displayed a diverse presence in the rhizodermis and in the treated roots all of the rhizodermal cells were labelled with the fluorochrome (Fig. 3B,F).

**Figure 4:**
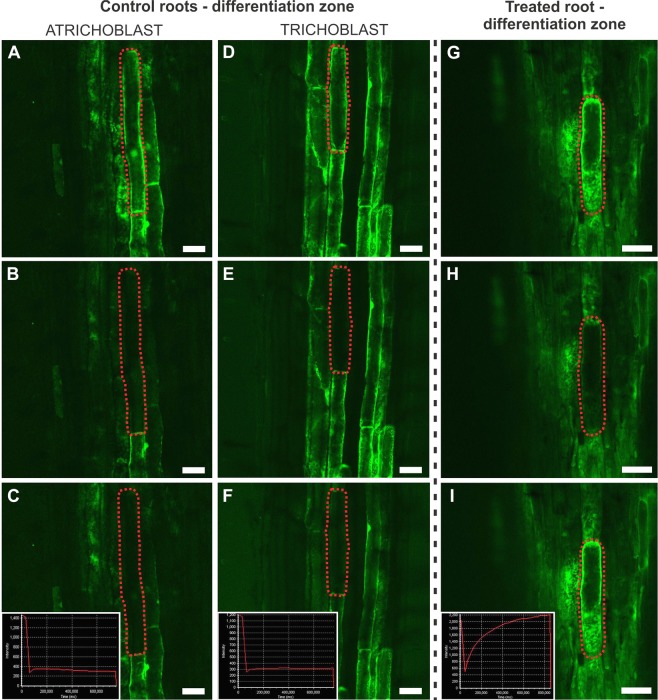
CLSM images of the fluorescence recovery after photobleaching (FDA staining) the rhizodermal cells of the control (**A**–**F**) and AuNPs-treated (**G**–**I**) roots in the late elongation/early differentiation zone. A lack of symplasmic communication between the rhizodermal cells in the control roots (FRAP was observed in the trichoblast cells: **A** – before photobleaching, **B** – just after photobleaching and **C** – about 10 min after photobleaching; atrichoblast cell: **D** – before photobleaching, **E** – just after photobleaching and **F** – about 10 min after photobleaching). The presence of symplasmic communication in the treated roots: **G** – before photobleaching, **H** – just after photobleaching and **I** – about 10 min after photobleaching (the cell that was photobleached is outlined by a red dotted line). Each section is representative of five biological replicas; in total 45 roots were analysed. Insets on **C**, **F** and **I** are representative charts of the fluorochrome intensity during FRAP. Scale bars = 50 µm.

The symplasmic communication between the rhizodermal cells in the differentiation zone was monitored using a confocal laser scanning microscope (CLSM) and FRAP (fluorescence recovery after photobleaching) using the FDA fluorochrome (Fig. 4; Table 2). In most cases of the control roots (Table 2), no recovery of the fluorescence was observed in the elongation/differentiation zone regardless of the cell type (trichoblast and atrichoblast), which had been photobleached (Fig. 4A–F; the cell types were determined based on their length). In the AuNPs-treated roots, the recovery of fluorescence was detected in most of the cells that had been photobleached (Fig. 4G–I; Table 2). These results indicate that in the elongation/differentiation zone, the root hair cells and non-hair cells of the control plants were symplasmically isolated from their neighbouring cells. By contrast, the cells were symplasmically connected in the same root zone in AuNPs-treated plants.

**Table 2:** Fluorescence recovery after photobleaching (FRAP) in rhizodermal cells of the control and AuNPs-treated roots.

Root	Control roots				50 µg/ml AuNPs-treated roots	
	Trichoblasts		Atrichoblasts			
	N^a^	%^b^	N	%	N	%
1	8	0.0	7	0.0	5	80.0
2	7	14.3	6	16.7	5	100.0
3	11	18.2	11	9.1	9	100.0
4	7	0.0	8	0.0	10	80.0
5	7	0.0	11	9.1	7	85.7

^a^number of evaluated cells, ^b^percent of cells to which the fluorochrome did return after photobleaching.

### Effect of AuNPs on the ultrastructure of rhizodermal cells

**Figure 5:**
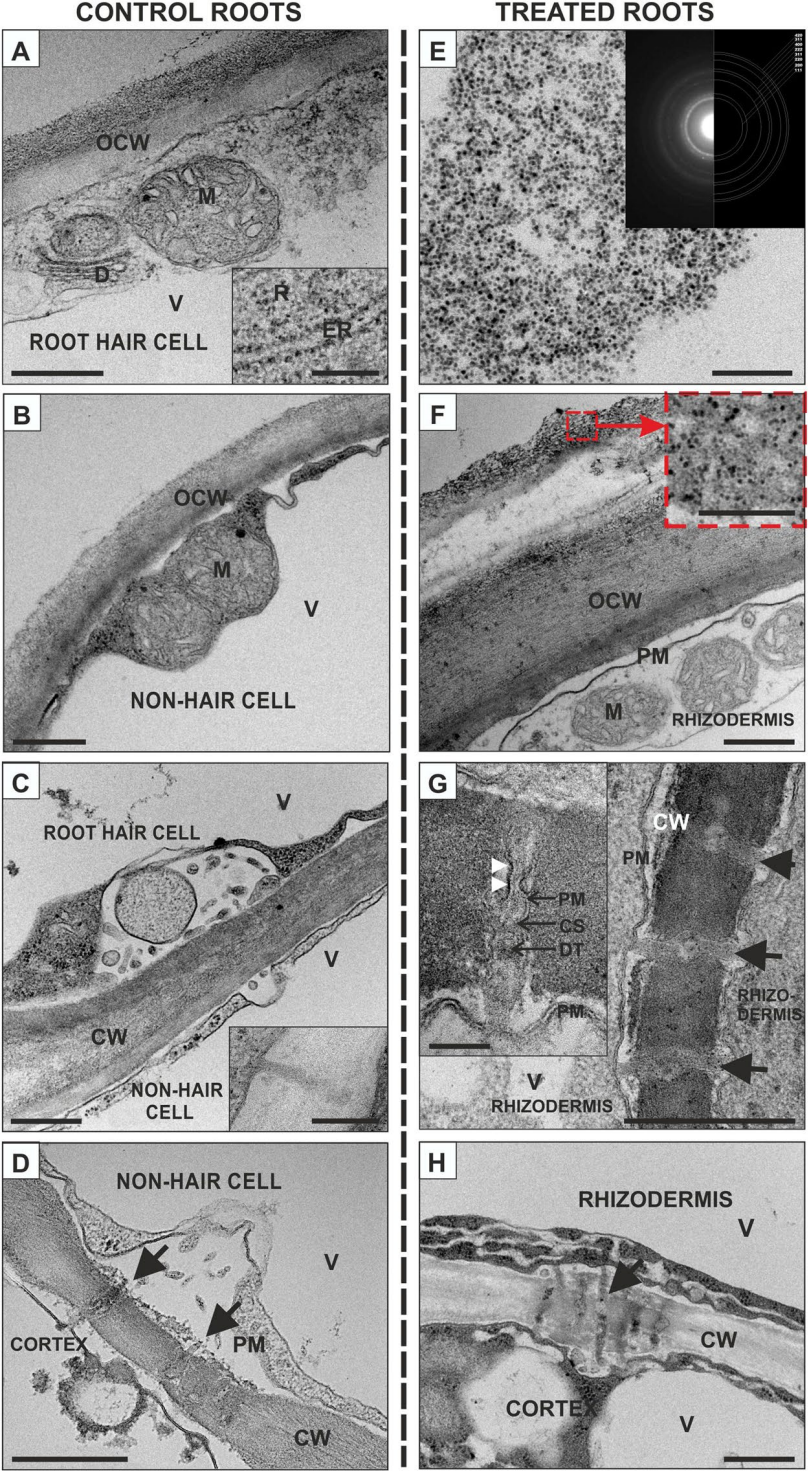
Representative ultrastructure of the rhizodermis cells from the differentiation zone of the control (**A**–**D**) and AuNPs-treated (**F**–**H**) roots from the barley seedlings (cross sections). **E** – TEM image of the AuNPs that were used in the presented studies and the diffraction pattern that was recorded with the corresponding theoretical rings for the Au structure (inset). The presented diffraction patterns were acquired using the Selected Area Electron Diffraction technique (SEAD). Using a specific selected area aperture, the region of interest in TEM image mode was selected and the corresponding diffraction patterns were recorded using a CCD camera. The selected area electron diffraction patterns for polycrystalline materials (or aggregation of NPs) has a circular character. A circle′s diameter is characteristic for crystalline materials because they correspond to the interplanar distances of a crystal lattice. Based on a circle’s position, it is possible to identify the studied material. The red simulated rings correspond to the theoretical positions of the circles that were calculated based on the Au crystal structure. The selected area diffraction patterns with overlaid simulated rings that are presented confirm the presence of AuNPs in the observed images. CW – cell wall; CS - cytoplasmic sleeve; D – dictyosome; DT – desmotubule; ER – endoplasmic reticulum; M – mitochondrion; PM – plasmalemma; R – ribosome; OCW – outer cell wall; V – vacuole; arrows – plasmodesmata; white arrowheads indicate some protein structures that are bound to plasmalemma of the PD. Each section is representative of: control – 8 roots; 25 µg/ml AuNPs – 6 roots; 50 µg/ml AuNPs – 6 roots. Scale bars: A-D, F-H = 500 nm; A, C insets = 200 nm; F, G insets = 100 nm.

An ultrastructural analysis of the rhizodermal cells of the control and treated roots (50 µg/ml AuNPs) in the differentiation zone was also performed (Fig. 5A–H) in order to answer the question of the ultrastructure of root cells and PDs. Non-hair cells differed in the thickness and ultrastructure of the outer periclinal walls, and there was a marked tendency for the increased vacuolisation. Both types of rhizodermal cells of the control roots contained a large central vacuole and well-developed organelles, including numerous mitochondria, ribosomes and endoplasmic reticulum in a very thin cytoplasmic layer along the intact cell wall (Fig. 5A–D). Although PDs were detected in all of the walls that were investigated, it was difficult to find them between the hair and non-hair cells (Fig. 5C), and numerous PDs were present between the non-hair cells and the cortex (Fig. 5D). The detected PDs were simple and, in most cases, three and four grouped together (Fig. 5D). In all cases it was possible to detect plasma membrane and desmotubule of a PD (Fig. 5D). Some PDs were extended in the central cavity regions.

The studies of the treated roots began with an analysis of a AuNPs solution (Fig. 5E). The diffraction pattern confirmed that the corresponding theoretical rings were in accordance with the Au structure (Fig. 5E inset). AuNPs were observed along the outer periclinal wall of the rhizodermis (Fig. 5F). Moreover, it was observed that the AuNPs passed through the outer layer of the cell wall, which was characterised by a loosened fibrillar structure (Fig. 5F inset). The cytoplasm of the rhizodermal cells was electron lucent (Fig. 5F) or electron dense (Fig. 5G) and filled with numerous organelles with a large number of mitochondria and vacuoles (Fig. 5F). In some cells, the cytoplasm which was located close to the cortex cells, a smooth endoplasmic reticulum that had a dilated, electron lucent lumen was observed (Fig. 5G). In the treated roots, the PDs were detected between the rhizodermal cells and between the rhizodermis and the cortex cells (Fig. 5G,H). The analysed PDs were simple and very often three to five (or even more) were grouped together (Fig. 5G,H). In the PDs between the rhizodermal cells, the central cavity regions were wide and in the PDs between the rhizodermal/cortex cells, electron-dense material was detected (Fig. 5G,H). The mean plasmodesmata diameter in AuNPs-treated roots was larger (54.3 nm; *P* < 0.001) than in control group (35.1 nm) (Fig. S3). We also calculated frequency of PD (PF) in the anticlinal cell wall of rhizodermis. The analysis showed significant differences in PF between control and AuNPs-treated roots as well as between different walls in the control root (walls between hair cell/hair cell, hair cell/non-hair cell and non-hair cell/non-hair cell) (Fig. S4).

### Changes in cell wall composition in the rhizodermis of the roots subjected to AuNPs treatment

Modifications in the cell wall chemistry may be a marker of changes in the external and internal environment. Moreover, these alterations may affect symplasmic transport since PDs are embedded in the cell wall. Thus, we decided to investigate the distribution of two pectic and two AGPs (arabinogalactan proteins) epitopes in the rhizodermis of control and treated roots (with high concentration of AuNPs).

**Figure 6:**
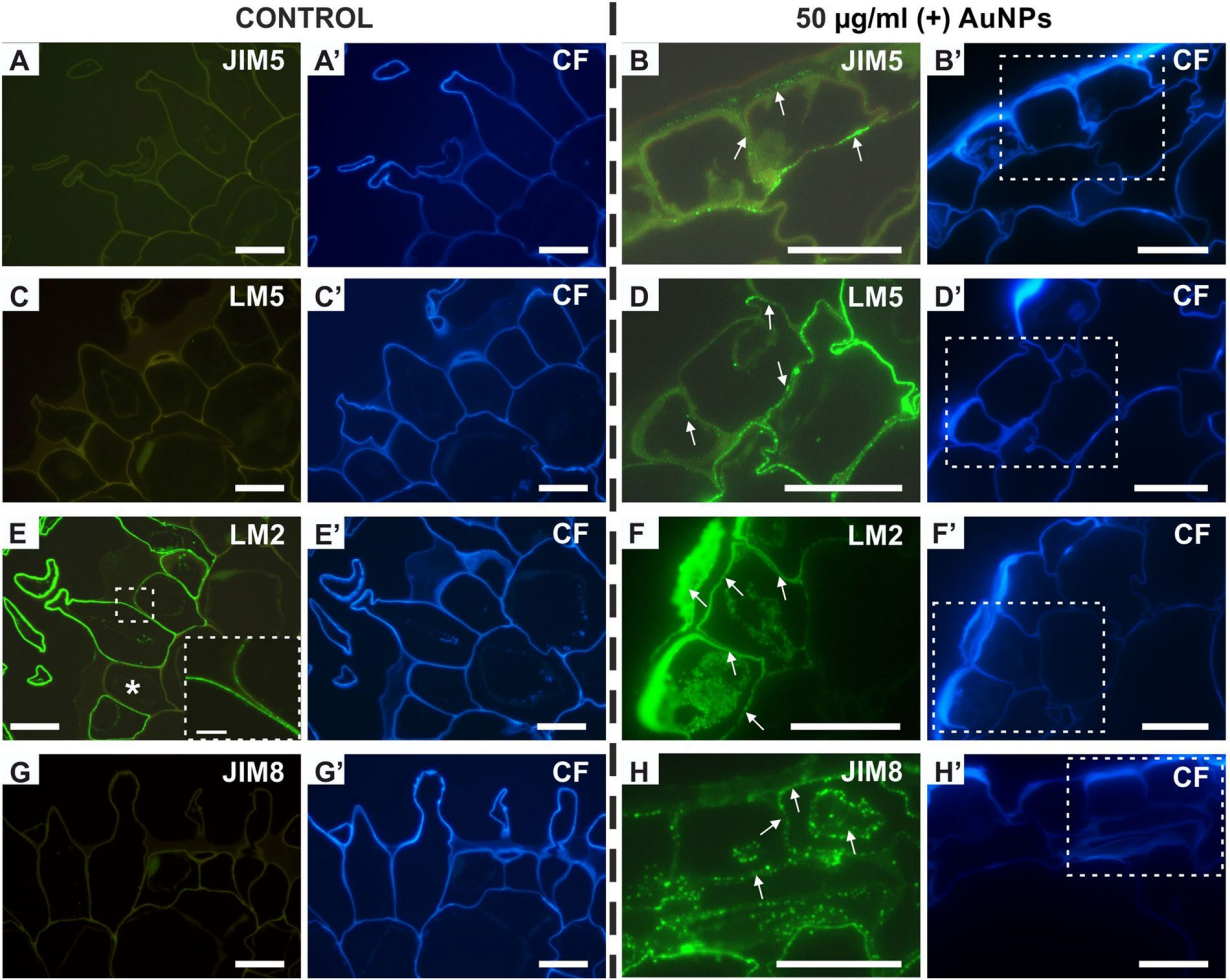
Immunofluorescent detection of the pectic (**A**–**D’**) and AGP (**E–H′**) epitopes in the rhizodermal cells from differentiation zone of the control (**A,A′,C,C′,E, E′,G,G**′) and treated (high concentration) roots (**B,B′,D,D′,F,F′,H,H′**). **B,D,F,H** are magnifications of areas marked by dotted line on **B′,D′,F′** and **H′**, respectively. Asterisk indicates the lack of the LM2 signal in non-hair cell of control root. Each section is representative of 15 roots. Scale bars: A-H′ = 20 µm; insets = 5 µm.

The pectic epitope that was recognised by the JIM5 antibody (low methyl-esterified HG) was not detected in the rhizodermal cells of the control roots (Fig. 6A,A′). In the roots that had been treated with nanogold at a high concentration, this epitope occurred in the outer and inner periclinal cell walls as well as in the anticlinal walls of the rhizodermal cells (Fig. 6B,B′). Although the LM5 galactan epitope was not observed in the control roots (Fig. 6C,C′), in the treated roots the signal was observed in the inner periclinal wall and in some regions of the anticlinal wall of the rhizodermis (Fig. 6D,D′). The most abundant presence of this epitope was observed in the outer layer of the cortex cells (Fig. 6D,D′). In the control roots the AGP epitope that was recognised by the LM2 antibody was detected in the walls of most of the rhizodermal cells (Fig. 6E,E′) (the signal was not detected in some of the non-hair cells; Fig. 6E, asterisk). In the treated roots, this epitope occurred more abundantly in the rhizodermal walls, especially in the outer periclinal wall (Fig. 6F,F′). Moreover, it was also detected in the cytoplasmic compartments (Fig. 6F,F′). The JIM8 epitope was not present in the rhizodermis of the control roots. This epitope (in the form of a punctate signal) was present in the rhizodermis as well as in the outer layer of the cortex cells of the treated roots. The epitope was distributed in the cell walls and in the cytoplasmic compartments (Fig. 6H,H′).

## Discussion

The influence of abiotic factors, e.g. metal toxicity on the development of plants including cell differentiation was shown in the case of the effect of metal on the morphology, histology, biochemistry, physiology, genetic and molecular levels^3,40^. For trichoblasts and atrichoblasts, it has been shown that the arrangement of the microtubules and microfilaments in these two types of cells was changed comparing to the control roots, at least in the case of some metals^40^. This implies that abiotic factors can affect the differentiation processes that occur in the rhizodermis during the development of the trichoblast and atrichoblast phenotype. Thus, the results presented here, which show the effect of NPs on the differentiation of the rhizodermis that leads to the hairless phenotype. Correlation of these phenomena with the remodelling of symplasmic transport add a new fact to the knowledge about the impact of abiotic stressors on plant growth and development. The lack of root hair cells may not only affect root growth but may also affect the growth of whole plants since the hair cells participate in the absorption of the phosphorus uptake from the soil^8,41,42^.

The results presented here indicate that the development of a hairless phenotype of barley roots may be an explanation (among others that have been described in the literature) for plant decay in the presence of AuNPs. Our observation suggests that this type of abiotic stressors may change the mechanisms that control cell differentiation, thus leading to a decrease in the surface area of a root system that can participate in the uptake of water and nutrients by plants^43^.

### Symplasmic transport and cell differentiation

The harmful effect of NPs on plant growth and development is well documented^2^ and the different mechanisms that lead to this effect have been studied and postulated^40^. However, to date, no information about the involvement of NPs in the remodeling of the symplasmic communication is available. The results presented here indicate that the distribution of symplasmic transport fluorochromes differed in the control and AuNPs-treated roots. The obtained results provide some information about the symplasmic communication between the root zones and tissues, especially in relation to trichoblasts/hair cells and atrichoblasts/non-hair cells and the development of the hairless phenotype. Namely, in the late elongation/early differentiation zone in the treated roots, the rhizodermal cells were symplasmically connected while in the control roots they were symplasmically isolated. This suggests that the remodeling of the symplasmic movement may block the differentiation of the hair cells.

The symplasmic communication between plant cells consists in the presence and functionality of PDs between neighbouring cells^26,44,45^. It is well documented that the process of plant cell differentiation depends, among others, on the symplasmic isolation or limitation in the symplasmic connection of cells^25,26,46^. It has been postulated that before beginning the differentiation programmes, cells or groups of cells restrict the symplasmic communication with the neighbourhood^20,26,45,47^. The correlation between symplasmic communication and root development suggests that the specification of cell fate and organ root formation depends on the movement of signals through the PDs^20,30^. It has been shown that symplasmic communication/isolation is critical for the initiating and positioning of the lateral roots meristems in Arabidopsis^46^. In the case of hair and non-hair cells differentiation, it was shown that the transcription of one-repeat Mybs (the gene family that is involved in root epidermal patterning) was found to predominately occur in the non-hair cells and that the proteins appear to move through the PDs and accumulate in the root hair cells^38,48^. The studies on the effects of nutrient scarcity and excess on symplasmic signaling in the Arabidopsis root revealed that changes in PDs permeability differ depending on nutrient stress. Reduction in the movement through PDs and in the root growth was observed in iron excess, whereas copper excess caused increase in PD permeability. These findings were correlated with decreases or increases in the levels of callose which regulate cell-to-cell connectivity^49^. These results showed that the response related to symplasmic signaling to different abiotic stress can vary depending on the dose and stress factor. Our results confirm this finding since AuNPs caused increase of movement through PD which is not a common response. This can be associated with changes in activities of callose synthases and β-1,3-glucanases which regulate callose deposition.

The only information about the involvement of symplasmic communication in rhizodermal cell differentiation comes from an analysis between the epidermal cells of *Hordeum vulgare* L., ‘Karat’, which have a normal root hair development, and two hairless mutants^31^. The results that were obtained indicated that symplasmic communication was limited during root hair differentiation in the parental variety, whereas the epidermal cells in the root hairless mutants were still symplasmically connected. This implies that in the hairless mutants, the lack of the limitation of symplasmic communication was correlated with the mutant phenotype. The results presented here indicate that the rhizodermal patterning into hair and non-hair cells can be changed by AuNPs. The development of a hairless phenotype may result from disorders and changes in symplasmic communication that block the differentiation of root hair cells. Certainly, this hypothesis must be verified for other species, other types of root epidermis patterning and other NPs. It is worth mentioning that hair cells were also not detected in *Lolium multiflorum* roots that had been treated with AgNPs^50^.

### Changes in PDs frequencies and diameter under the AuNPs influence

The number of PDs or their frequencies is calculated primarily for tissues involved in the exchange of assimilates in the case of leaves, shoots and roots^51–56^. Therefore, it is difficult to compare the results obtained by us with the literature data. The only available data point to the statements that PDs in rhizodermal cells of terrestrial plants are extremely rare^57^ and that the PDs number between rhizodermal cells is smaller compared to other root tissues^45,58^.

For many years, the plasmodesmogram, which is a two-dimentional diagram of PD frequencies, was used to describe the integrative action of tissues and organs basing on the assumption that the greater the frequency of PDs, the greater the potential for symplasmic communication is^59^. However, the recent findings that PDs are channels with different functional diameters, a correlation between PDs frequencies and the rate of intercellular communication is disputable^59^. Nevertheless, we made an attempt to determine PDs frequencies between rhizodermal cells in the anticlinal walls in AuNPs-treated (50 µg/ml) and control roots. In the case of the control roots, the number and frequencies of PD in anticlinal walls between hair cell/hair cell, non-hair cell/non-hair cell and hair cell/non-hair cell were calculated (Fig. S4). Such estimations have shown the differences in PD frequencies between control and treated roots.

In general the PD diameter is between 25–40 nm, but PD diameter of 50 nm and even 80 nm have also been reported^60–62^. Our analysis showed that the PD diameter for the control roots is within the limits given by the literature. However, the PD diameter in the treated roots is much larger. Such widening of PD diameter was described in cases of plant reaction to diverse external factors^63–65^. In the mentioned above cases PD widening is connected with an increase in symplasmic connectivity^35^. Therefore, it is reasonable to suppose that widening of PD is a plant response to harmful agent which is AuNPs and may cause PDs more permeable.

### Root epidermis patterning

The root epidermis consists of two type of cells, hair cells (developed from trichoblasts) and non-hair cells (developed from atrichoblasts). These two types of cells are arranged within the rhizodermis in a species-dependent pattern^66^. In vascular plants, three types of hair and non-hair cells distribution are recognized^67^: in type 1, the hair cells are randomly distributed, while in type 2, an asymmetric cell division is responsible for hair and non-hair cell differentiation/distribution and in type 3, in Arabidopsis, which is the best described and analysed, it is dependent on a cell position^68^. In grasses, two types of rhizodermal patterning have been documented^7,11,69^. Within the rhizodermis, every cell is capable of differentiation into root hair cells, or in the case of an unequal division, only the shorter cells emerge as root hair cells. For barley, it was shown that a second pattern operates^7,69^. In our studies, we used the same cultivar of *Hordeum vulgare* L. as Marzec *et al*.^7^ and we also detected cells with different lengths in the control roots in which the shorter ones differentiated into root hairs, thus confirming the previous results. The mean cell lengths of the tricho- and atrichoblasts that we observed are similar to those previously stated^7^. It is noteworthy that in the case of the *rhl1.b* hairless mutant^7^, the length of the rhizodermal cells is greater than in the case of the roots that had been treated with AuNPs. However, in this case, shorter rhizodermal cells are characterised by a hairless phenotype. A decrease in the length of the root epidermal cells was described for maize roots that was growing under the salt stress^70^ and in Arabidopsis^71^, which may suggests that harmful factors, independent of their character, affect the cell differentiation of roots. The differences in the root cell sizes (both in diameter and length) and the root histology (among others increased number of metaxylem elements and atypical divisions of rhizodermal and cortex cells) between control and treated with the highest AuNPs concentration that were detected in presented studies indicate that the NPs that had been used also affected these parameters.

### AuNPs treatment affect cell wall composition

Changes in the chemical composition of the cell walls are a marker of changes in the external and internal environment. Therefore, an immunohistological analysis of the presence/distribution of selected AGP and pectic epitopes was also performed. The obtained results showed that among the studied epitopes, only the epitope that is recognised by the LM2 antibody was detected in the control roots, whereas the treated roots were characterised by the presence of all of the analysed epitopes (JIM5, LM5, LM2, JIM8).

The cell wall is mainly composed of cellulose, but the presence of proteins, pectins and hemicelluloses cause the wall to be a complex network of chemical components, and that the qualitative and quantitative changes correspond with various developmental processes and manifest themselves in the reactions of plants to biotic and abiotic stresses^72,73^. AGPs and pectins are postulated to be involved in many developmental processes, including the reaction of the plant cell wall to changes in the external and internal factors^74–81^.

The results on the distribution of pectic and AGPs epitopes are difficult to compare with literature data because studies on the chemical composition of the rhizodermal walls of monocotyledonous plants are very limited. Most studies on the role of individual cell wall components on the growth of rhizodermal cells, trichoblasts and atrichoblasts and the development of root hairs have been carried out on dicotyledonous^82,83^.

From all of the analysed pectic and AGPs epitopes in control roots, only the AGP epitope that is recognised by the LM2 antibody was detected. Similar results were described for the barley cv. Karat and the hairless mutant *rhl1.b*^7^. The similarity of the results presented here with the results that have been obtained for the hairless mutant in the aspect of AGPs indicates that the observed changes in symplasmic communication are also correlated with the composition of the cell wall.

Because PDs are embedded in the cell wall, the chemical composition of the wall may affect symplasmic transport^20^. There is no doubt that callose is involved in the regulation of cell-to-cell movement^84^. It was also suggested that cellulose and pectins, including the modification of pectins^85^, may be involved in this regulation^86^. The results indicate that in the cells of the roots that had been treated with AuNPs, low methyl-esterified HG and (1 → 4)-β-D-galactan occurred in the outer and inner periclinal walls, and in trace amounts in the anticlinal walls. Moreover, it is known that the composition of the cell walls around the PD is different from other wall regions^85^. Most of the information comes from studies on the presence of callose, which is a factor that controls the PD diameter and thus its functionality^20^. Knowledge about the pectins and cell wall proteins in the neighbourhood of the PD is very scarce^87^. As was pointed out, the PD proteome identifies the candidates that are implicated in pectin biosynthesis and/or remodelling. The results presented here, which show differences in the presence of the pectic and AGPs epitopes in the rhizodermal cells of the control and treated roots, indicate the involvement of changes in the presence of these compounds in the regulation of symplasmic cell-to-cell communication^87^.

## In conclusion

The results presented here showed that AuNPs influenced the morphology, histology, ultrastructure, chemical composition of cell wall as well as cell differentiation of roots. The results suggest that AuNPs blocks the differentiation of root hair cells, leading to a hairless phenotype in barley, through a mechanism that involve the remodelling of symplasmic cell-to-cell communication.

## Material and Methods

### Material

*Hordeum vulgare* L. cultivar Karat was used for the studies (caryopses were derived from the collection of the Faculty of Biology and Environmental Protection of the Department of Genetics at the University of Silesia and were provided through the courtesy of professor Iwona Szarejko). The barley caryopses were processed according to a method that was described earlier^88^. Briefly, the surfaces of the caryopses were sterilised by immersing them in a 20% sodium hypochlorite solution for 20 minutes and subsequently washing them in sterilised distilled water three times for five minutes after which they were left in the distilled water for imbibition (24 h at room temperature). The seeds were germinated in a hydroponic solution using glass tubes that were sealed with Parafilm (in a growth chamber under conditions of a 16 h photoperiod, 20 °C and 180 μE m^−2^ s^−1^ of light) for seven days.

The control plants were grown in a 1/16-strength Hoagland medium and the experimental plants were grown in a solution of 5 nm of positively charged AuNPs at two different concentrations: 25 μg/ml and 50 μg/ml.

### Methods

#### Nanoparticle characterisation

Gold (5 nm spheres) nanoparticles (AuNPs) were obtained from nanoComposix Europe, the Czech Republic. The surfaces of the AuNPs were modified using branched polyethyleneimine (BPEI) containing the amino groups that cause the formation of positively charged AuNPs. The diameter of the NPs of the BPEI coating was between 1–2 nm. The AuNPs solution had an intense red shade.

#### Morphological and histological analyses

The morphology of the roots was studied using an SMZ 1500 stereomicroscope (Nikon, Tokyo, Japan) equipped with a Nikon Digital DS-Fi digital camera.

Sample fixation, dehydration and embedding for the histological analysis were conducted in accordance with the method for ultrastructural studies (described below). Sections were stained with 0.05% (w/v) Toluidine blue 0 (TBO; Sigma-Aldrich, St. Louis, MO, USA) according to a procedure that was described earlier^35^.

#### Data analysis

Measurements of the roots length, meristematic zone length, cell wall length, cell diameter and PD diameter were carried out using ImageJ software (version 1.49; http://imagej.nih.gov/). For the measurements of the meristem length, the border between the meristem and the elongation zone was measured according to Kirschner *et al*.^89^. The diameter of the cells was measured along four designated radii at the level of the differentiation zone (the number of repeats and the number of cells are given in Sup. Mat.). Data were subjected to a one-way analysis of variance (ANOVA) and the effect of AuNPs treatment was considered significant at the significance level *P* < 0.05. Diameters of PD were measured from electronograms (mean of 55 (control) and 46 (AuNPs-treated) PDs at their largest dimensions in the longitudinal view). Means of PD diameter were compared using the Student t-test. The effect of AuNPs on hairy root development was assessed using the statistical test for significance level between two proportions. Differences between means were compared using Tukey’s honest significant difference (HSD) test implemented in the Statistica v.12 software.

#### Calculation of PDs frequencies

PDs frequencies were calculated according to Ma and Peterson (2001)^53^ with the use of following formula: Fw = N/[L(T + 1.5 R)].

*N* is the number of PDs along the wall; *L* is the length of analysed wall (anticlinal cell wall of rhizodermis); *T* is the thickness of sections (0.07 µm), and *R* is the PD radius. To obtain *N* and *L*, more than 100 walls were randomly sampled from 10 non‐serial ultrathin sections (which were cut from 5 roots). On each wall, *N* was counted directly in the microscope. Length of the walls were calculated based on 105 walls for 50 µg/ml AuNPs and 18, 45 and 39 walls between hair cell/hair cell, hair cell/non-hair cell and non-hair cell/non-hair cell respectively.

**Table:** 

		L [µm]	N	R [µm]	T [µm]	Fw
	50 µg/ml AuNPs	14.99	96	0.054	0.07	78.66
Control	hair cell/hair cell	28.86	15	0.035	0.07	9.86
	hair cell/non-hair cell	27.71	50	0.035	0.07	34.24
	non-hair cell/non-hair cell	26.52	23	0.035	0.07	16.46

#### Fluorochromes of the symplasmic tracers

Symplasmic communication was examined using the following fluorochromes. HPTS (8-hydroxypyrene-1,3,6-trisulfonic acid, trisodium salt) and solutions of HPTS (Thermo Fisher Scientific, Waltham, MA, USA, H-348, MW = 520 Da) were prepared by dissolving 5 mg of the fluorochrome in 1 ml of demineralised water^28,29^. The fluorochrome distribution was analysed using an epifluorescence microscope (Nikon Eclipse Ni, Tokyo, Japan). The fluorochrome was excited using a B-2A filter (excitation wavelength 450–490 nm; the emission of the HPTS was collected at 520 nm). The roots were placed into the fluorochrome solution and cut off under the surface of the solution. After 30 minutes, the roots were washed several times in demineralised water, placed on microscopic slides, covered with a cover glass and analysed using a fluorescence microscope.

In total, the roots from four different seedlings were analysed and the experiments were repeated five times for both the control and the treated plants (the specific number of analysed roots/cells is given in the figure legends). The figures (photographs) were edited using the CorelDrawX7 graphics program.

#### Confocal microscopy – FRAP

Fluorescein diacetate (FDA; Sigma-Aldrich, Cat. No. F7378, Poznan, Poland) was prepared according to Marzec *et al*.^31^ with some modifications. A stock solution that was prepared in acetone (5 mg ml^−1^) was dissolved in demineralised water (0.5 ml per 24.5 ml) in order to obtain the FDA working solution.

Fluorescence recovery after photobleaching (FRAP) was used to monitor the movement of the FDA within the epidermis of the barley root in the late elongation/early differentiation zone. For fluorescence analyses, a 20x UPlanFLN objective lenses (NA 0.5) was used in an Olympus FV1000 confocal system (Olympus, Warsaw, Poland). The pre- and post-bleaching events were imaged using 6% power of a 488-nm line from an argon-ion laser (Melles Griot BV, Didam, the Netherlands); emission was detected using a 505–530 nm band pass filter. For the photobleaching of the fluorescence in a single cell, 90% power of the laser 405-nm line was used for 30 s. The roots that were still attached to the whole plant, were exposed to FDA for 15 min and thoroughly washed with water before microscopy studies. Analyses were performed within the root part of the late elongation/early differentiation zones, which was determined based on the cell length between the elongation zone and the differentiation zone in which no signs of root hair differentiation was present. Each probe (control and 50 µg/ml AuNPs) consisted of the roots from four kernels, all of which were analysed and five biological replicates were performed for this experiment. Photo-documentation was performed just before FRAP, just after and 10 min after photobleaching. In the preliminary experiments it was found that prolonging time after FRAP did not result in the return of fluorochrome to the cells, thus the set time was 10 minutes, which agrees with analyses that were previously carried out^31^.

The frequencies of fluorochrome movement between the different root cells were scored and assessed according to the method described by Kim *et al*.^90^. Briefly, the number of cells to which the fluorochrome did not return after photobleaching was compared to all of the cells that were tested. For the treated roots, the number of cells to which fluorochrome did return after photobleaching was compared to all of the cells that were tested. Afterwards, the percentage of these ratios was calculated (Table 2).

#### Transmission Electron Microscopy (TEM)

Samples for the control and treated roots were fixed in 2.5% glutaraldehyde (Sigma-Aldrich) and 2.5% paraformaldehyde (Polysciences, Warrington, PA, USA) in a 0.05 M cacodylate buffer (CB; Sigma; pH 7.2) and kept for 24 h at 4 °C. Next, the samples were washed in CB, postfixed in 1% osmium tetraoxide (OsO_4_; Serva) in CB for 3 h, rinsed in the same buffer and dehydrated in a graded series of ethanol and gradually embedded in Epon resin (Polysciences, Warrington, PA, USA) according to a method that was described earlier^88^. For the TEM analysis, ultrathin sections, 70 nm thick, were obtained using a Leica EM UC6 ultramicrotome and were deposited on carbon-coated copper grids (200 mesh, Electron Microscopy Science, Hatfield, PA, USA). The sectioned grids were stained with a saturated solution of uranyl acetate (Polysciences, Warrington, PA, USA) in 50% ethanol for 15 min and 0.04% lead citrate agents (Sigma) for 10 min. The samples were analysed using a Jeol JEM-3010 (300 kV) High Resolution Electron Microscope (HRTEM) equipped with an EDS (Energy Dispersive Spectrometry) spectrometer and a Gatan 2k × 2k Orius^TM^ 833 SC200D CCD camera.

#### Immunohistochemistry

The immunlabelling analyses were performed on a cross section from the differentiation zone of the control roots and the roots that had been treated with nanogold (only roots that were growing in the 50 μg/ml AuNPs solution were analysed because of the prominent changes in the rhizodermis pattern). The plant material was fixed in a mixture of 4% PFA and 1% GA in phosphate buffered saline (PBS, pH = 7.2) overnight at 4 °C. Subsequently, the samples were washed three times at PBS followed by dehydration in increasing ethanol concentrations [10, 30, 50, 70, 90, 100% (v/v) EtOH in distilled water 2 × 30 min each step] and gradually embedded in LR White resin (Polysciences, Warrington, PA, USA). Next, the samples were cut into 1.5 µm thick cross sections using an EM UC6 ultramicrotome (Leica Biosystems). The sections were placed on glass slides that had been coated with poly-L-lysine. The immunostaining procedure was performed exactly as described by Betekhtin *et al*.^91^. The specific primary monoclonal antibodies (Plant Probes) that were used in the study are presented in Table 3. The secondary antibody was AlexaFluor 488 goat anti-rat (Jackson Immuno Research Laboratories, West Grove, PA, USA). The negative controls were performed by omitting the primary antibody, thus obtaining no fluorescence signal in the control set of the sections. All of the images were taken using a Nikon Eclipse Ni-U epifluorescence microscope equipped with a Nikon Digital DS-Fi1-U3 camera with corresponding software (Nikon, Tokyo, Japan) and a maximum excitation wavelength of 490 nm (AlexaFluor 488). The photographs were edited using the CorelDrawX7 graphics program.

**Table 3:** List of the primary monoclonal antibodies that were used for the immunofluorescence studies.

Epitope	Antibody	References
*Pectins*		
JIM5	Low methyl-esterified HG	Clausen *et al*.^92^
LM5	(1 → 4)-β-D-galactan	Jones *et al*.^93^
*Arabinogalactan proteins (AGPs)*		
LM2	β-linked GlcA	Yates *et al*.^94^; Smallwood *et al*.^95^
JIM8	Arabinogalactan	Pennell *et al*.^96^

## Supplementary information

Supplementary Information
