# Supplementary material for: The development of a hairless phenotype in barley roots treated with gold nanoparticles is accompanied by changes in the symplasmic communication
Source: Sci Rep. 2019 Mar 18;9:4724. doi: 10.1038/s41598-019-41164-7 (PMC6423127; doi:10.1038/s41598-019-41164-7)

**The development of a hairless phenotype in barley roots treated with gold nanoparticles is accompanied by changes in the symplasmic communication**

Anna Milewska-Hendel^1*^, Weronika Witek^1^, Aleksandra Rypień^2^, Maciej Zubko^3^, Rafał Barański^4^, Danuta Stróż^3^ & Ewa U. Kurczyńska^1*^

^1^Department of Cell Biology, Faculty of Biology and Environmental Protection, University of Silesia in Katowice, 28 Jagiellońska Street, 40-032 Katowice, Poland

^2^Laboratory of Microscopy Techniques, Faculty of Biology and Environmental Protection, University of Silesia in Katowice, 28 Jagiellońska Street, 40-032 Katowice, Poland

^3^Institute of Materials Science, Faculty of Computer Science and Materials Science, University of Silesia in Katowice, 75 Pułku Piechoty Street 1a, Chorzów, 41-500, Poland.

^4^Institute of Plant Biology and Biotechnology, Faculty of Biotechnology and Horticulture, University of Agriculture in Krakow, Al. 29 Listopada 54, 31-425 Krakow, Poland

*E-mail addresses of the corresponding authors: [anna.milewska@us.edu.pl](mailto:anna.milewska@us.edu.pl), [ewa.kurczynska@us.edu.pl](mailto:ewa.kurczynska@us.edu.pl)

**Table S1.** Quantification of root phenotypes in control and AuNPs in different concentration.

|  | N | Frequency of hairless roots (%) | *P* |
| --- | --- | --- | --- |
| Control | 33 | 0 | - |
| 25 µg/ml AuNPs | 37 | 24,32 | 0.002 |
| 50 µg/ml AuNPs | 36 | 88,89 | < 0.001 |

*The effect of AuNPs on hairy root development was assessed using the statistical test for significance level between two proportions.* *N – number of evaluated roots; P – significance level.*

**Figure S1.** Root length in the control and AuNPs-treated roots.

Dot – mean value, box – mean ± std. error, whiskers – mean ± 1.96 std. error.

**Figure S2.** Cell diameter in the control and AuNPs-treated roots.

Dot – mean value, box – mean ± std. error, whiskers – mean ± 1.96 std. error.

|  |  |
| --- | --- |
|  |  |
|  |  |

**Figure S3.** Diameter of plasmodesmata in rhizodermal cells in control and 50 µg/ml AuNPs-treated roots.

Dot – mean value, box – mean ± std. error, whiskers – mean ± 1.96 std. error.

**50 µg/ml AuNPs**

|  | PD diameter [nm] |
| --- | --- |
| Control | 35,13 (n=55) |
| 50 µg/ml AuNPs | 54,24 (n=46) |

*Means of plasmodesmata diameter were compared using the Student t-test; n – number of evaluated PDs.*

**Figure S4.** Frequencies of PD between rhizodermal cells in the control and 50 µg/ml AuNPs-treated roots. Red – number of PD per walls between marked cells; green – PD frequencies; HC – hair cell; NHC – non-hair cell.


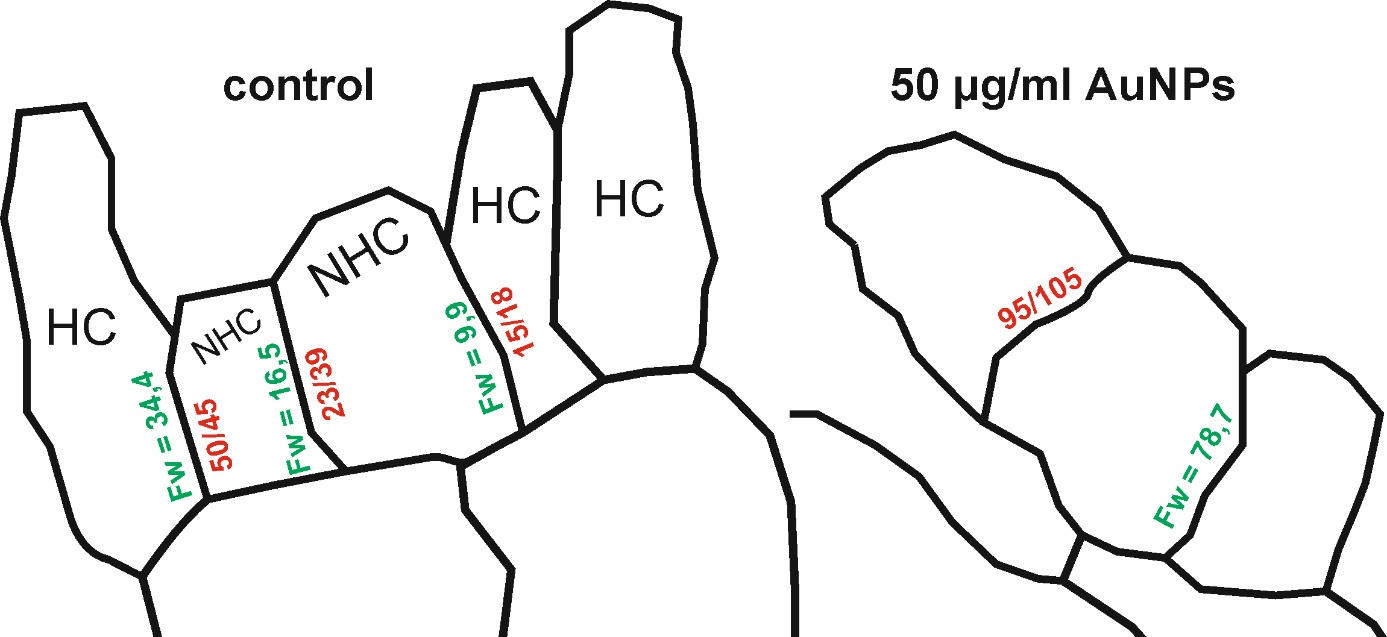

Supplement: Supplementary file 1 — Supplementary Information [file 41598_2019_41164_MOESM1_ESM.docx]
